# Decolonising global health: transnational research partnerships under the spotlight

**DOI:** 10.1093/inthealth/ihaa073

**Published:** 2020-11-09

**Authors:** David S Lawrence, Lioba A Hirsch

**Affiliations:** Department of Clinical Research, Faculty of Infectious and Tropical Diseases, The London School of Hygiene and Tropical Medicine, London, UK; Botswana Harvard AIDS Institute Partnership, Gaborone, Botswana; Department of Public Health, Environments and Society, Faculty of Public Health and Policy, London School of Hygiene and Tropical Medicine, London, UK

**Keywords:** authorship, decolonisation, ethics, global health, transnational research partnerships

## Abstract

There are increasing calls to decolonise aspects of science, and global health is no exception. The decolonising global health movement acknowledges that global health research perpetuates existing power imbalances and aims to identify concrete ways in which global health teaching and research can overcome its colonial past and present. Using the context of clinical trials implemented through transnational research partnerships (TRPs) as a case study, this narrative review brings together perspectives from clinical research and social science to lay out specific ways in which TRPs build on and perpetuate colonial power relations. We will explore three core components of TRPs: participant experience, expertise and infrastructure, and authorship. By combining a critical perspective with recently published literature we will recommend specific ways in which TRPs can be decolonised. We conclude by discussing decolonising global health as a potential practice and object of research. By doing this we intend to frame the decolonising global health movement as one that is accessible to everyone and within which we can all play an active role.

## Introduction

There are increasing calls to decolonise aspects of science, and global health is no exception. What it means to decolonise global health is not always well explained or understood and to some the act itself may seem too ill-defined, obscure or daunting for it to be achievable. Using the context of clinical trials conducted through transnational research partnerships (TRPs) as a case study, the purpose of this article is to demonstrate that a multidisciplinary approach, combining the practical experience of a research physician with the critical perspective of a social scientist, can be applied to critique aspects of global health research. We will draw particularly on experience from the continent of Africa, but aspects of this review will apply to broader contexts. We focus particularly on randomised controlled trials, which are, despite criticism,^1^^,^^2^ regarded as producing the most rigorous data for an intervention and, possibly because of this, where TRPs are commonly found. Specifically, we historicise and contextualise three aspects of TRPs (participant experience, expertise and infrastructure, and authorship) to lay out specific ways in which TRPs build on and perpetuate colonial power relations before suggesting specific ways in which we can work towards more equitable TRPs. By doing this, we intend to frame the decolonising global health movement as one that is accessible to everyone and within which we can and should all play an active role. We refrain here from offering a normative or static definition of what decolonising global health means and accept, following Tuck and Yang,^3^ that real decolonisation needs to take place outside academia and needs to be led and abide by the principles of indigenous communities. Although we primarily focus on decolonisation, we also recognise the intersectional vulnerabilities that disproportionately affect women and junior researchers (of colour) within global health.^4^

### The decolonising global health movement

When working in the field of global health research one is constantly exposed to, and even complicit in, the power imbalances that exist between researchers in high-income countries (HICs), researchers in low- and middle-income countries (LMICs) and the research participants we work with.^5^ These inequalities largely derive from colonialism and are frequently the subject of debate within the field.^6^ Indeed, the very notion of global health and global health research have been subject to the label of ‘scientific colonialism’.^6^^,^^7^ Against this background, various ‘decolonising global health’ (DGH) movements have emerged at universities in the last few years.^8^^–^^10^ Importantly, these movements are often student-led and include high proportions of students from LMICs and their diasporas. Where we work, at the London School of Hygiene and Tropical Medicine, the DGH movement has started exploring concrete ways in which global health teaching and research can overcome its colonial past in terms of representation, research and funding processes. These movements, which have emerged predominantly within universities in the Global North, are only one of many manifestations that critiques of our current global health system have taken. As pointed out by DGH movements, we have to be mindful to listen to and learn from the people at the receiving end of global health interventions. In this article we want to draw on the important and innovative work of the DGH movement to think through and provide suggestions for the decolonisation of TRPs.^4^

### The importance of global health research

Clinical trials generate important knowledge and there is a need for well-conducted clinical trials to take place in sub-Saharan Africa. In fact, there has been long-standing criticism surrounding the general neglect of the region in terms of clinical research. Roughly 80% of all clinical trials are conducted in HICs^11^ and diseases of relevance to HICs are investigated in clinical trials seven to eight times more often than those whose burden lies in LMICs.^12^ Criticism continues concerning the representation of different populations in research and this is exemplified by the disproportionately low number of individuals from LMICs who are recruited into clinical trials, despite the often higher burden of disease.^13^ For example, one review found that in the context of human immunodeficiency virus (HIV), only 31% of all prevention or treatment trials prior to 2009 were conducted in LMICs despite these countries representing 99% of the mortality associated with HIV infection.^14^ The underrepresentation of LMIC populations in medical trials, apart from being seemingly unfair, has important consequences for how we understand and measure health. Biomedical interventions in LMICs have, since the advent of tropical medicine, relied on the assumption that European white bodies are the normative gauge of health.^15^ As with women and children, who have historically been excluded from medical trials,^16^^,^^17^ the exclusion of diverse, non-European bodies impacts who treatments and medications are designed for and is likely to disadvantage the health of non-white European male bodies.^18^ Research in LMICs is therefore essential but must be designed to benefit the local population. Foreign characterisations of LMICs as suitable locations for high-risk or ethically dubious research that can benefit individuals in HICs, as was recently proposed by researchers trialling a coronavirus vaccine, must be rejected.^19^

### Transnational research partnerships

Clinical trials in LMICs are most commonly conducted through TRPs. These are cooperative pieces of research conducted by a combination of research institutions from different countries. In the context of global health, these partnerships are almost always between institutions in both LMICs and HICs. The premise of these partnerships is that institutions can collectively pool expertise, infrastructure and resources to deliver a high-quality outcome. The number of partners may be as small as two, but can be much larger. In an attempt to quantify the scale of these partnerships, a study from 2005 identified all published HIV treatment and prevention trials conducted in sub-Saharan Africa between 1987 and 2003 and focused on funding, geographical reach and authorship.^20^ A total of 77 published trials that recruited patients from across 18 countries were included. The main funders were government agencies outside of Africa in 56% of trials and the pharmaceutical industry provided either full or partial funding to 44%. Funding from African government or non-governmental organisations contributed to 5 of 77 trials but were not the sole funders of any. In addition, this review found that the chief investigator was resident in Africa in only 25% of the trials, with the majority being from outside the continent, including the USA (30%) and the UK (10%), among other countries. An update to this work reported more recent trials conducted between 2004 and 2008 and identified no notable change in these trends over time.^21^ These geographical dimensions highlight the continued dependence of African members of TRPs on countries and institutions in the Global North. We now turn to analyse this dependence in more detail using the examples of participant experience, expertise and infrastructure, and authorship.

## Analysis

### The participant experience

Clinical trials are only possible thanks to the willing participation of research participants and they should be at the centre of all debate and discussion. To date, there exists no published research that has specifically explored the perspective of participants in LMICs when it comes to the structure of global health research. During clinical trials administered through TRPs, research participants are not directly asked for their thoughts on this subject. Here we aim to provide two examples that shed light on how the experience of clinical trials in sub-Saharan Africa is permeated by colonial history and colonial power relations: rumours and informed consent.

A large portion of the ethnographic work exploring participant experience of research in LMICs has elicited data concerning rumours, most commonly in the context of blood stealing. In East and Central Africa, the historical basis for such rumours has been linked to the violent and extractive practices of colonial medical officers in the 19th and 20th centuries.^22^ Today these rumours may be dismissed as expressions of ignorance or simply as being related to ‘culture’, but numerous social scientists have described them as forms of popular resistance.^23^^–^^25^ In research within healthy volunteer studies, the generation of rumours about research studies and institutions is particularly prevalent when poor outcomes such as severe disability or death occur and, even in the absence of any clear link to the research study, there is often an apportioning of blame. These rumours often contain local interpretations of medical research ethics, especially related to the problems of resource transfers and flows of value. It has been argued that rather than ignoring rumours, engaging with them could enrich medical research ethics debates and improve relations between medical researchers and study communities.^25^

Although the phenomenological interpretations of rumours provide an important window into the perspectives of research participants, further research exploring their experience of trials administered through TRPs is required. Individuals ought to be given the opportunity to articulate directly their perspective of how clinical trials are conducted rather than solely being interpreted from other observations (most commonly those of foreign researchers). This will also be enhanced by increased diversity within research teams and locally-led research protocols that can effectively elicit and accurately interpret research findings.

It is beyond the scope of this article to outline all the ways in which international research standards may not suit particular contexts, and in-depth research has been published elsewhere,^26^^,^^27^ but let us now briefly turn to one example of informed consent. Informed consent, a product and signifier of conducting ethical research according to European standards, often falls short of successfully translating into varied research contexts outside of Europe and North America. As it is, we continue to fall short of ensuring a full and equivalent understanding of what giving consent means in the context of TRPs. These issues are magnified by the increase in genetic analyses taking place within trials and the storage of genetic material that often takes place outside the countries in which clinical trials occur.^28^^,^^29^ There are already documented cases of research misconduct and exploitation in this field.^29^^,^^30^ Further research is essential to understand how participants experience and interpret research ethics in a changing world.

### Expertise and infrastructure

In the context of HIV research, particularly concerning HIV treatment and prevention, large, multisite trials are often conceived and designed by international research networks based in HICs. These groups then subsequently identify country leads at each site who they will then work with to recruit and train teams of researchers for that specific site. In these cases, protocol development, the design of standard operating procedures, trial oversight and data management often occur remotely to the sites, which means that local researchers implement the trial but do not necessarily gain the skills required to later run their own trials.^31^ In addition, most research studies involve increasingly complex analyses of specimens—for example, genetic analysis, which can only currently be performed in a limited number of state-of-the-art laboratories that are most often found outside of sub-Saharan Africa. The location of medical infrastructures is hugely important, because infrastructures are often needed to turn knowledge into expertise and capacity, both of which are requirements for individual career progression. They are also necessary to attract future funding and the leadership of clinical trials. Over time, the development of advanced laboratory infrastructure in some LMICs has increased their competitiveness when it comes to clinical research.^32^

There is huge promise in TRPs, but there is also significant potential to create and perpetuate power imbalances both between and within individual institutions. Most commonly, chief investigators based in HICs apply to funding bodies, also often based in HICs, and collectively steer the research agenda in one direction or another. These researchers are typically employed by research institutions from HICs who measure the performance of their staff based on research funding and publications, which may distract them from the more subjective outputs of capacity building.^7^ If these strategic funding decisions are not made in consultation with local researchers, there is a potential to overlook the most pressing research questions for the population as well as the opportunity to build capacity in that country.^33^ The most extreme forms of this type of work are exemplified in the frequent reports of ‘fly-in, fly-out’ or ‘parasitic’ researchers who parachute into LMICs for short periods of time to collect data and samples before returning home to publish their findings, often bypassing local researchers and research needs entirely and, according to one focus group discussion, leaving institutions feeling like ‘poor prostitutes’.^34^^–^^36^

Funding of clinical trials is hugely influential and there are examples of best practice whereby funding is channelled to researchers and institutions based in sub-Saharan Africa, with a focus not just on research outputs, but on institutional and individual capacity building. The European and Developing Countries Clinical Trials Partnership (EDCTP) is a notable example here.^37^ The EDCTP funds clinical trials that address the most pressing public health needs within a country or region, while also providing additional funding to build capacity within research institutions. This approach requires a significant investment of time and resources, but such a model could create a future wherein TRPs occur solely between African institutions. South–South TRPs have been made more difficult given the slashing of health budgets of countries in the Global South through structural adjustment programmes in the 1980s and 1990s.^38^ Going forward and where possible, governments need to integrate research funding into their healthcare budgets while balancing the demand to provide healthcare services with immediate benefits. Member states of the African Union, through the Africa Health Strategy 2007–2015, have committed to allocating 2% of their healthcare budget to research.^39^ Although a situational analysis of this programme in 2017 found that target had not been met, this commitment was renewed in the Health Research and Innovation Strategy for Africa 2018–2030.^40^

The way in which success is quantified in global health research also needs to change. When individual performance is based on successful grant applications and authorship, this distracts from other meaningful outputs, such as mentorship and capacity building. International researchers are therefore, often understandably, guilty of prioritising their own research outputs rather than helping to develop the skills of their colleagues, which is an equally constructive and often more meaningful use of time. Therefore funders and research institutions need to place greater emphasis on this work in their appraisal of individuals or risk perpetuating the idea of research as a white male domain.^41^

### Authorship

Another way to explore how TRPs disproportionately benefit researchers in the Global North is to look at authorship. The number of first or final author papers that an individual has is used to gauge their prominence in the field and is often the first port-of-call for funding bodies when reviewing a grant application or institutions when considering a promotion. The issue of authorship is complex and there exist established guidelines that outline what constitutes an author or a contributor to a piece of research. In large clinical trials that employ hundreds of staff it is often impossible to list each individual, particularly when some journals place a limit on the number of authors, although this is less common than it used to be.^42^ Including only individuals meeting the definition of an author may technically be fair and author positioning may be a true reflection of the workload undertaken and the ‘scientific input’ provided, however, it highlights further that the beneficiaries of global health research are often those based in high-income institutions. As recent studies have shown, researchers from LMICs are often ‘stuck in the middle’ when it comes to global health authorship resulting from international partnerships, further widening the divide between those researchers who benefit from TRPs (predominantly white and European or North American) and those who do not.^43^^,^^44^ Abimbola's recent editorial on the foreign gaze in global health authorship makes an equally important point.^45^ He explains the difference between the foreign and local gaze and asks us to question what the foreign gaze actually contributes to a holistic understanding of health in LMICs. These are deeply necessary questions and conversations to have and to which we hope to contribute here.

In addition, there is evidence suggesting that global health research is impacted by guest authorship, which is adding authors who did not contribute substantially to the work, and ghost authorship, which is omitting authors who have contributed substantially to the work. A survey and interview–based study solicited responses from researchers based in LMICs who were presented with various scenarios about authorship, redundant publication, plagiarism and conflict of interest and asked for their opinions and experiences of each.^46^ In this study, 77% of participants reported the use of guest authorship in their institution and 41% reported occurrences of ghost authorship. There is not currently enough evidence to truly determine whether this is more common in global health research than in any other discipline, although this has been suggested,^47^ and no truly comparative work has been done in high-income settings to enable a fair comparison. However, this does demonstrate a widespread unfairness in how academic work is recognised in this setting.

Authorship will always be important in research, but it is vital to factor in the contribution made by all parties and advocate for joint authorship and the most inclusive authorship policy possible, ensuring that any scientific contribution, no matter how small, is recognised. In addition, the roles that result in the more prestigious authorship positions need to be available and accessible to a more diverse group of researchers. We must also recognise to what extent we, as researchers and practitioners from or based in the Global North, have benefitted from a global health system built on colonial medicine, which continues to replicate colonial power dynamics in infrastructure, expertise and authorship.

## Discussion

### Decolonising TRPs

How can one work towards decolonising TRPs that, at their core, further dependence? Should we be speaking of partnerships at all? Surely the aim of decolonising TRPs should be to negate the need, motivation or opportunity for certain individuals and institutions to be involved at all. There are significant aspects of global health research that need to change and this is a process that needs to take place over time, rather than overnight, so as not to jeopardise the real benefits to health that result from this research. And it should start now.

We all have a responsibility to create an open environment whereby it is safe to discuss this issue. It is often easier for researchers in HICs to discuss neo-colonial aspects of global health research but it is far more intimidating for the majority of African researchers to do the same, particularly among their international collaborators. This reluctance is yet another colonial aspect of global health. There are intellectual decolonisation movements occurring across Africa, with the University of Cape Town being a notable example. But within TRPs there is a need for spaces and forums for debate across the continent where African and international researchers are able to have frank, open discussions about these power imbalances and develop solutions going forward. International researchers also need to embrace what is often an inconvenient truth. The London School of Hygiene and Tropical Medicine, where we work, has made a start in this regard, forming a working group that is exploring the school's historical links to colonialism and exploring how a world-renowned academic institution can undergo transformation. But each setting is nuanced and these conversations are required at the country and institutional level.

Working on the assumption that TRPs will not disappear overnight and that international researchers are likely to remain engaged in research in Africa for some time, it becomes important to focus on how they navigate through this space. This is increasingly relevant, as the trend seems to be for institutions in HICs to increasingly build links with those in LMICs. International researchers are a heterogeneous group, some of whom are respectful of institutional culture and reflective on their position within it, whereas others are less so. We all need to be culturally and racially literate when it comes to global health research and this needs to happen as early in our careers as possible. Medical schools and research institutions have a duty to generate discussion concerning the complexity of global health research and to develop, in partnership with their collaborators, guidelines for the responsible conduct of research in LMICs. It is not acceptable for researchers to assume that because they are from an international institution that they are more knowledgeable, that their work should take priority or that they deserve the premium position on papers. This is a particular risk when individuals are new to the research world or the country in which they are conducting research. A prolonged, sustained period of conducting research in a country will always be far preferable to a fly-in, fly-out approach.

### Research and decolonisation

There is a need for further research into TRPs. With an increased appreciation of the potential harms and benefits of TRPs there is an urgent need for an exploration of their impact on all stakeholders. This includes ongoing quantification and monitoring of key indicators related to the three domains we have discussed as well as in-depth qualitative studies. In particular, no previous original research exploring TRPs from the perspective of researchers from both HICs and LMICs has been published. We need to understand how research participants themselves experience the research process within these partnerships. This is the partial focus of an ongoing ethnographic study being conducted by D. S. Lawrence and colleagues (ClinicalTrials.gov: NCT04296292).

There is also a need to focus on decolonisation as the subject of research. Any research into this topic needs to be carefully planned to ensure that it does not (re)create the existing power imbalances and biases it is trying to address and is aware of its limitations. We all need to do the work to make global health truly global. This means giving voice to the global majority in global health authorship, listening to the experiences of research participants and making sure that clinical trials increasingly take place in the countries and regions whose health problems they work to alleviate. First and foremost, it means checking our own privilege and realising that as practitioners and researchers based in and affiliated with institutions in the Global North, we continue to benefit from a global health system built on colonial medicine. All research and commentary will be restricted by the lived experience of the individual researchers, this article included. Decolonising global health presents an opportunity to make global health more inclusive and work towards health justice. Let's get to it.
